# Video image of genital melanosis provides strong evidence to support identification of a sexual offender

**DOI:** 10.1007/s12024-021-00364-2

**Published:** 2021-04-05

**Authors:** Atsushi Yamada, Toshio Demitsu, Naoka Umemoto, Osamu Kitamura

**Affiliations:** 1 Department of Legal Medicine, Kyorin University School of Medicine, Tokyo, Japan; 2 Department of Dermatology, Jichi Medical University Saitama Medical Center, Saitama, Japan

**Keywords:** Forensic image evidence, Dermatology, Genital melanosis, Sexual offense

## Abstract

A man in his thirties was suspected of committing a sexual offense against a young girl. A video on his mobile telephone provided the only evidence. Photographs obtained from the video showed male genitalia in two views, with the penis in both views exhibiting unique pigmentation. We appraised this case with the cooperation of dermatologists, who diagnosed the pigmentation as male genital melanosis, a relatively rare disease, which matched that on the suspected perpetrator’s penis. Photographs obtained from the video were thus decisive evidence of sexual offense and identified the perpetrator.

## Introduction

Establishing identity is a fundamental role of a forensic examination, with personal characteristics such as hair color, surgical scars, and tattoos serving as clues to the identification [1]. In addition, several reports have indicated the potential use of basic dermatology in forensic examinations [2, 3]. This article presents a case of sexual offense successfully resolved via close collaboration with dermatologists.

## Case presentation

**Fig. 1:**
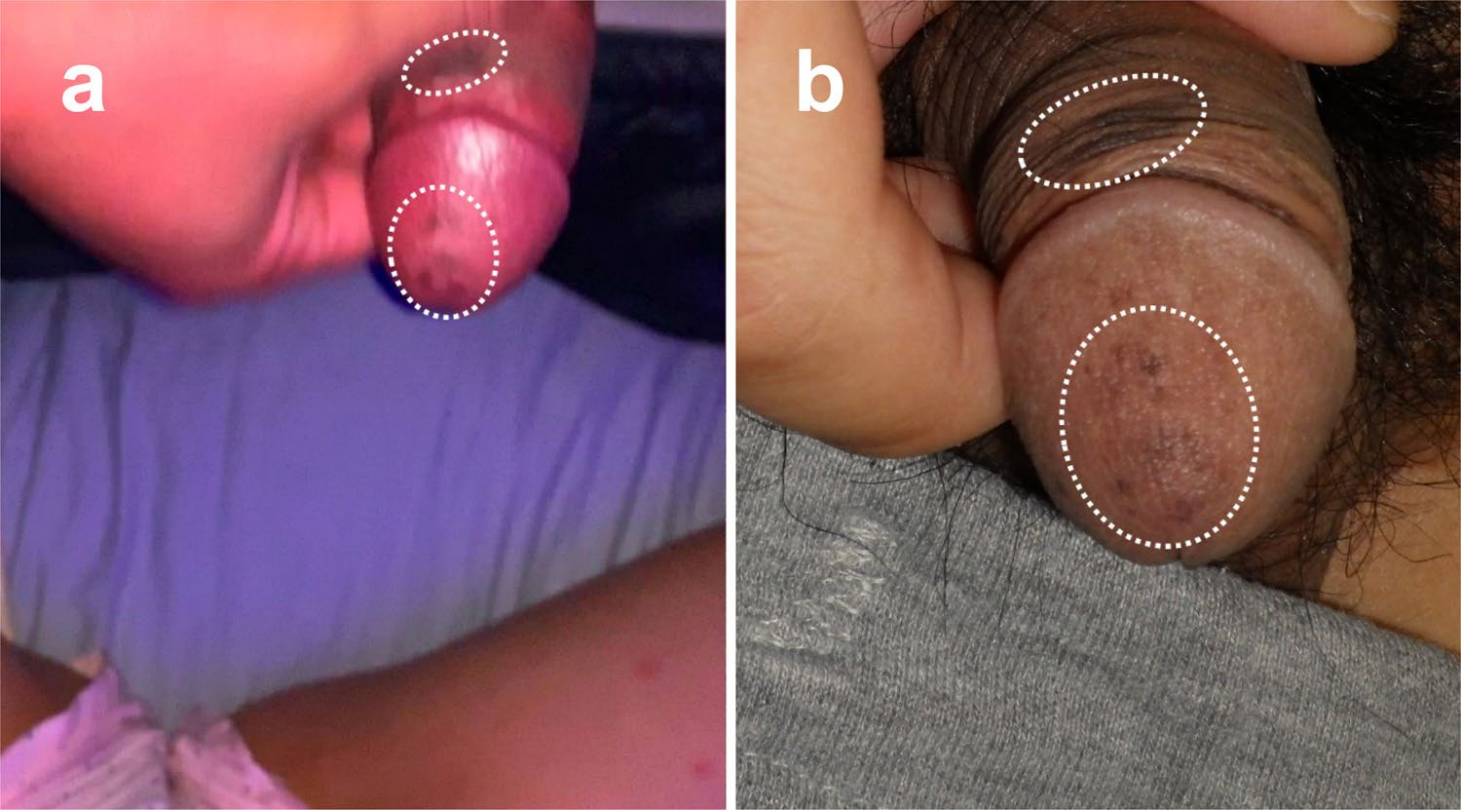
**a** Screen capture of the video found in a mobile phone of the suspect. **b** Suspect holds the penis at the same position under interrogation. Comparison of the two images (**a** and **b**) shows the generally homologous distribution of the pigmented skin lesions (indicated by dotted ovals)

A man in his early thirties was suspected to have committed a sexual offense against a young girl, although he denied the accusation. The police found a video on his mobile phone in which the crime was recorded. Unfortunately, the video showed only the attacker’s genitals (Fig. 1a). The suspect declared that a third party had broken into his house and used the telephone to record himself during sexual intercourse. The police, however, noticed peculiar skin pigmentation on the suspect’s penis that was also apparent in the video (Fig. 1b). At this point in the investigation, the police asked our Legal Medicine Department to appraise the case. We then consulted dermatologists to identify what the skin pigmentation represented and if it was useful for proving that the penis displayed in the video belonged to the suspect.

**Fig. 2:**
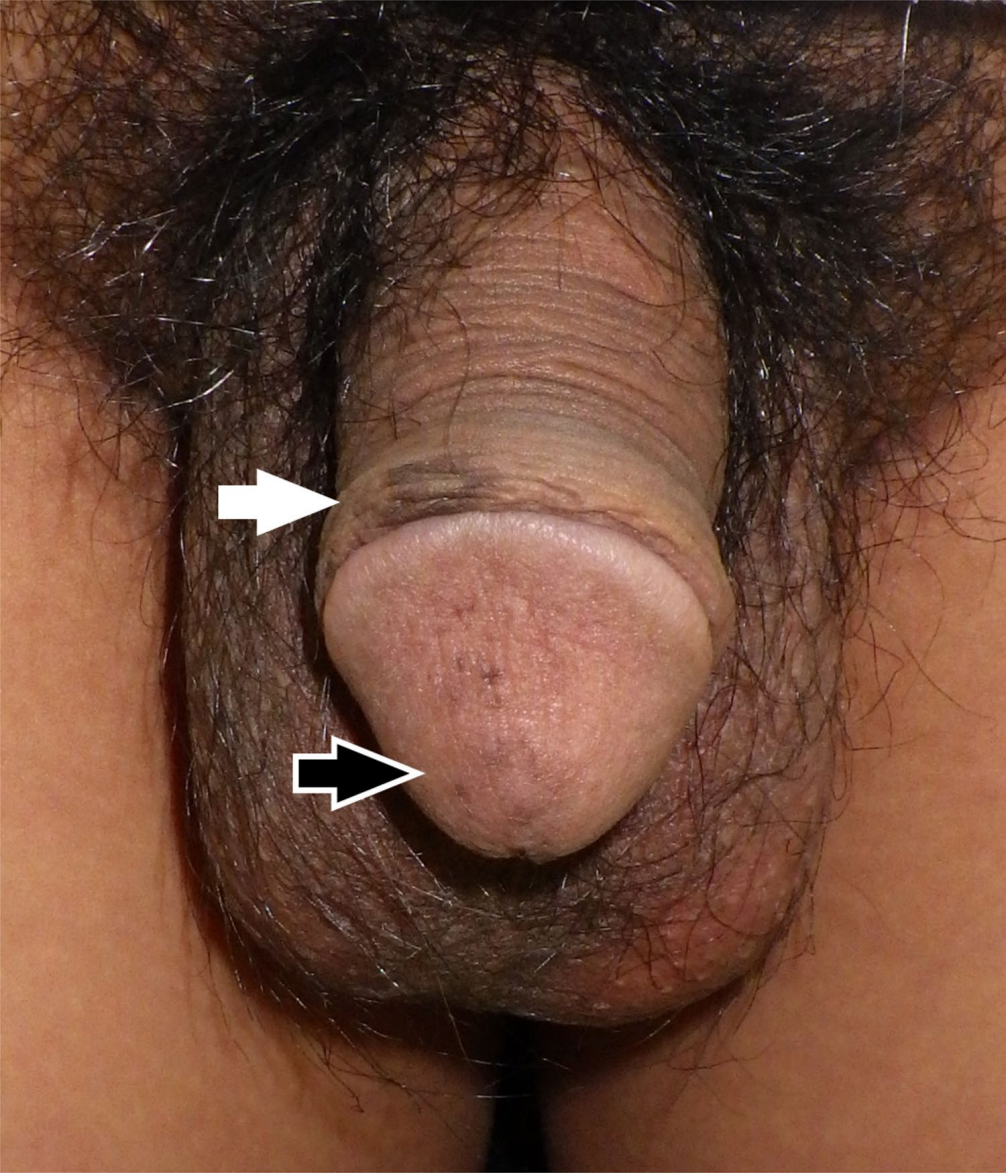
Gross appearance of the suspect’s genitalia. Multiple gray-to-black macules are seen in the glans penis (black arrow). A patch of the same nature is also present in the distal portion of the prepuce (white arrow)

During the interrogation, we had obtained a screen capture of the video, and photographs were prepared of the pigmented penis. The dermatologists identified multiple sporadic macules with no scales, irregular borders, and varying shades of gray to black that were slightly dense at the tip of the glans penis. A gray-to-black single patch was also present in the distal portion of the prepuce (Figs. 1 & 2). The suspect declared no symptoms except the skin eruption, which, to his recollection, had been present with no marked change since puberty. A skin biopsy was not performed. Taking his clinical course into account, the dermatologists diagnosed genital melanosis and proved that the two penises in the images belonged to the same man. In the presence of this clinical evidence, the suspect confessed to the crime.

## Discussion

Genital melanosis in men is relatively rare, with an estimated incidence of 0.011% among dermatologic patients [4]. Because of the lack of subjective symptoms, however, the true prevalence of genital melanosis is thought to be slightly higher. Hence, this dermatologic finding could be useful for identifying individuals.

The decisive factor that made the suspect in this case admit guilt was not only the homology of penile pigmentation but also anxiety. In clinical practice, pigmented lesions of the genitalia always require exclusion of malignancy, represented by melanoma. Genital melanoma accounts for 8–10% of all genital malignancies and is the second most common genital cancer after squamous cell carcinoma [5, 6]. Although genital melanosis is thought to be a benign entity, some reports have suggested an association between genital melanosis and malignant melanoma [4, 7]. Over a 10-year follow-up in one study, 15% of patients with genital melanosis had a history of melanoma elsewhere in the body [4]. Therefore, by way of precaution, we recommended that the suspect visit the dermatology center for further evaluation, to which he agreed.

Characteristic penile skin eruptions in the video image in this suspect allowed us to establish his identify and facilitate his confession. Previously, the distribution patterns of melanocytic nevi have been successfully applied to assess individuals via forensic image comparisons [2]. Needless to say, dermatologists are indispensable for a precise diagnosis of nevi. They are professionally trained in visual examination, which results not only in an accurate diagnosis but also learning the patient’s background (i.e. physical condition, comorbidities, habits, and lifestyle). Hence, we suggest that it may be worthwhile to investigate the feasibility of integrating dermatologic theory into forensic evaluations.

## Data Availability

The data that support the findings of this study are available from the corresponding author, upon reasonable request.
